# 

**Published:** 1921-09-17

**Authors:** 


September 17, 1921] [Price Twopence

### The Hospital

The Workers' Newspaper of Administrative Medicine and Institutional Life, Administration, National Insurance and Health.

### CONTENTS

**Tuberculous Cripples** ... ... ... ... ... 407

**A Dream of Health** ... ... ... ... ... 408

**Approved Societies and Hospitals** ... ... ... 408
An Analysis of Seventy-three Appeals.

**Notes of the Week** ... ... ... ... .. 409
"Our Day" in London—The Leicester Celebrations—Welfare Supervision—Miners and Hospital Contributions—A Local Hospital Pageant—Indian Graduates' Midwifery—The Hampshire "Hospital Committees"—Rose Day and Race Days—The Royal Waterloo Hospital—A School Doctor on Daylight Saving—The Call of the Hospital—Welcome Bequests to Glasgow Hospitals.

**An Excellent Record** ... ... ... ... ... 411
The Metropolitan Asylums Board Report for 1920-21.

**Rays from the Eye** ... ... ... ... ... 412

**The Mental Hospitals Association** ... ... ... 412

**The Road to Co-ordination** ... ... ... ... 413

What the Peace Programme Has Done.

**The Institutional Library** .. ... ... ... 414

Radiographic Technique—Mother and Child—Œdema and Nephritis—An Atlas of Normal Labour.

**The Public Well-Being** ... ... ... ... ... 415

The Welfare of Migrants: World's Y.W.C.A. Report.

**The Editor's Letter-Box** ... ... ... ... 416
The National Provident Scheme—The Battle of Jutland Film—Floors and Walls of Operating-Theatres.

**The Matrons' and Sisters' Department** ... ... 417
Nurses as the Pioneer New Women.
The Inspiration of Example.

**Round the Hospitals** ... ... ... ... ... 418

**Letters to a Nurse** ... ... ... ... ... 419

I.—Medical Manners and Mystery.

**The London Hospital** ... ... ... ... ... 420

Economies and Sources of Income.

**Matrons' and Sisters' Appointments** ... ... v

**The College of Nursing (Limited by Guarantee)** v

